# Hepatoprotective effects of hoveniae semen cum fructus extracts in ethanol intoxicated mice

**DOI:** 10.20463/jenb.2016.03.20.1.4

**Published:** 2016-03-31

**Authors:** Ilje Cho, Joowan Kim, Jaijun Jung, Soohyun Sung, Jongkyu Kim, Namju Lee, Saekwang Ku

**Affiliations:** 1 Aribio Central Research Institute, Aribio Inc., Sungnam-siRepublic of Korea; 2 Department of Sports Medicine, Jungwon University, Goesan-gunRepublic of Korea; 3 Department of Anatomy and Histology, Daegu Haany University, Gyeongsan-siRepublic of Korea

**Keywords:** Hoveniae, hepatoprotective, mouse, Nrf2, antioxidant

## Abstract

**[Purpose]:**

The objective of this study was to evaluate the hepatoprotective effects of Hoveniae Semen Cum Fructus extract in ethanol induced hepatic damages.

**[Methods]:**

Hepatic damages were induced by oral administration of ethanol and then Hoveniae Semen Cum Fructus extract was administered.

**[Results]:**

Following Hoveniae Semen Cum Fructus extract administration, body and liver weights were increased, while aspartate aminotransferase, alanine aminotransferase, albumin, *γ*-glutamyl transferase, and triglyceride levels in the serum, triglyceride contents, tumor necrosis factor -*α* level, cytochrome (CY) P450 2E1 activity in the liver and mRNA expression of hepatic lipogenic genes, and Nitrotyrosine and 4-HNE-immunolabelled hepatocytes were decreased. However, mRNA expression of genes involved in fatty acid oxidation was increased. Also, as a protective mechanism for hepatic antioxidant defense systems, decreased liver MDA contents, increased glutathione contents, increased dismutase and catalase activities were observed when compared to the ethanol control.

**[Conclusion]:**

Hoveniae Semen Cum Fructus extract favorably protected against liver damages, mediated by its potent anti-inflammatory and anti-steatosis properties through the augmentation of the hepatic antioxidant defense system by NF-E2-related factor-2 activation, and down-regulation of the mRNA expression of hepatic lipogenic genes or up-regulation of the mRNA expression of genes involved in fatty acid oxidation.

## INTRODUCTION

The liver is an important organ actively involved in metabolic functions and is a frequent target of a number of toxicants1. It is well known that a substantial increase in steatosis and fibrosis usually leads to potentially lethal cirrhosis of the liver in humans^2^. Alcohol-related disorders are one of the challenging current health problems with far reaching medical, social, and economic consequences. Long-term alcohol use potentially results in serious illnesses, including alcoholic fatty liver, hypertriglyceridaemia, cirrhosis, cardiovascular disease, and inflammation of the pancreas^3^. Although much progress has been made in understanding the pathogenesis of alcoholic liver disease, there is no effective therapy for the disease. Novel therapeutic targets that successfully correct the fundamental cellular disturbances resulting from excessive alcohol consumption are effective^4,5^. Alcoholic liver disease is a result of complex pathophysiological events involving various types of cells, such as neutrophils, endothelial cells, Kupffer cells, and hepatocytes, and a variety of injurious factors such as endotoxin, oxidative stress, cytokines, and proteases^6^. Accumulated evidence has demonstrated that oxidative stress, abnormal cytokine production, especially tumor necrosis factor (TNF), and steatosis play important etiological roles in the pathogenesis of alcoholic liver disease. Therefore, agents that have antioxidant, anti-inflammatory and anti-steatosis properties, particularly anti-TNF production and decreasing lipid accumulation, represent promising therapeutic interventions for alcoholic liver disease^4,5,7^.

Ethanol (EtOH) administration causes the accumulation of reactive oxygen species (ROS), including superoxide, hydroxyl radical, and hydrogen peroxide^8^. ROS cause lipid peroxidation of cellular membranes, protein and DNA oxidation, which results in hepatocyte injury^9,10^. The potential harmful effects of these oxidant species are controlled by the cellular antioxidant defense system^11^. Reduced glutathione (GSH) is the predominant defense against ROS/free radicals in different tissues of the body^12^. In addition, antioxidant enzymes, such as superoxide dismutase (SOD), catalase (CAT), glutamate cystein ligase (GCL) and Hemeoxygenase-1 (HO-1) are essential in both scavenging ROS/free radicals and maintaining cellular stability^13^. Under normal conditions, the reductive and oxidative capacities of the cell (redox state) favor oxidation. However, when the generation of ROS in cells impairs antioxidant defenses or exceeds the ability of the antioxidant defense system to eliminate them, oxidative stress results^14^. It is conceivable that agents with a hepatic antioxidant property would have therapeutic potential for alcoholic liver disease^4^. Oxidative stress and lipid peroxidation are predominantly generated through the induction of cytochrome (CY) P450 2E1 15. A key role for this enzyme in EtOH-induced liver injury has been demonstrated by its inhibition through chlormethiazole and by the finding that CYP450 2E1 knock-out mice do not show evidence of EtOH-induced liver disease^16^.

It is well established that increased reactive oxygen species and electrophiles induce a series of antioxidant genes via the activation of antioxidant response elements (AREs). ARE-driven gene expression is mainly regulated by NF-E2-related factor-2 (Nrf2), a member of the cap’n’collar family of bZIP transcription factors, which are essential transcription factors that regulate the expression of major antioxidant enzymes including glutathione S-transferase A1/2, hemeoxygenase 1, UDP-glucuronosyl transferase 1A, NAD(P)H:quinone reductase, and -glutamylcysteine synthetase^17^. Nrf2-knockout mice are characterized by susceptibility to oxidative stress, including acetaminophen, UV irradiation and carcinogens 18. Therefore, Nrf2 plays a role as a multi-organ protector in oxidative stress-related diseases^19^.

Another major consequence of EtOH metabolism is lipid accumulation in the liver. EtOH metabolism changes the NAD/NADH ratio, which has important consequences on fuel utilization in the liver, favoring the synthesis of fatty acids and inhibiting their oxidation 20,21. Sterol regulatory element-binding protein-1c (SREBP-1c) and peroxisome proliferator-activated receptor (PPAR) *α*, two nuclear transcription regulators controlling lipid metabolism, are involved in the development of alcoholic fatty liver^5,15^. EtOH administration activates hepatic SREBP-1c gene and its target genes: fatty acid synthase (FAS), stearoyl-CoA desaturase 1 (SCD1), and acetyl-CoA carboxylase 1 (ACC1), which promotes *de novo* fatty-acid synthesis^5,15^. It also increases the expression of genes for PPAR*γ* and diacylglycerol acyltransferase (DGAT) 2, which promotes triglyceride (TG) synthesis^5,15,22-24^. EtOH decreases the expression of mRNA encoding PPAR*α*, acyl-CoA oxidase (ACO) and carnitine palmitoyltransferase1 (CPT1), which leads to the inhibition of fatty acid oxidation^5,15,25^.

EtOH-mediated experimental liver damaged rodents have been used for detecting the hepatoprotective effects of various herbal extracts or their chemical components based on the changes of body and liver weights. Histopathology of the liver with blood chemistry like serum aspartate aminotransferase (AST), alanine aminotransferase (ALT), alkaline phosphatase (ALP), TG, *γ*-glutamyl transferase (*γ*-GTP), and albumin with hepatic TG contents, hepatic lipid peroxidative makers, mRNA expression of hepatic lipogenic genes or genes involved in fatty acid oxidation, and especially histopathological changes of hepatic parenchyma have been used as critical end points of EtOH-mediated hepatic damages in rodent models^4,5,15,26-30^.

Hoveniae Semen Cum Fructus (HSCF) is the dried peduncle of *Hovenia dulcis* Thunb. (Rhamnaceae). Various antioxidant based pharmacological effects of HSCF extracts have been reported including anti-adipogenic^31^, anti-fatigue^32^, neuroprotective^33^ and hepatoprotective^34,35^ effects. However, it seems more systemic evaluation of the hepatoprotective effects of HSCF extract with molecular targets is needed. In the present study, the beneficial potential of HSCF extract on subacute EtOH-induced hepatic damages in C57BL/6 mice was systemically investigated as well as the associated potent anti-oxidant, anti-inflammatory and anti-steatosis mechanisms.

## METHODS

### Preparations and administration of test materials

HSCF extracts (contains about 8.20ug/mg quercetin) were supplied by Aribio (Seoul, Korea) as a beige powder. HSCF was ground and extracted with hot water 2 times at 95°C for 4 hours then filtered and condensed using a rotary vacuum evaporator (EYELA N-1200B, USA). Finally, it was dried and standardized with dextrin using a spray drier (about 7.4ug/g quercetin). The HSCF extract was obtained as 26%. A reddish-yellow powder of silymarin was purchased from Sigma-Aldrich (St. Louise, MO, USA) as the reference drug. All test materials were stored at -20°C in a refrigerator to protect from light and humidity until used. In this study, 500 mg/kg was selected as the highest dose of the HSCF extract based on the clinical dosage in humans and 250 and 125 mg/kg were additionally selected as the middle and lowest doses with a common ratio of 2, respectively. HSCF extract (500, 250, and 125 mg/kg) and Silymarin (250 mg/kg) were suspended in distilled water and orally administered once a day after 1 hour of EtOH treatment for 14 days. In intact and EtOH control mice, equal volumes of distilled water were orally administered.

### Animals and experimental design

A total of sixty-three healthy male SPF/VAF Inbred C57BL/6J mice (6-wk old upon receipt; OrientBio, Seungnam, Korea) were used after acclimatization for 10 days. Animals were allocated five per polycarbonate cage in a temperature (20-25°C) and humidity (50-55%) controlled room. The dark light cycle was 12hrs long. Commercial rodent feed (Samyang, Seoul, Korea) and tap water were supplied *ad libitum*. All animals were treated according to the international regulations for the usage and welfare of laboratory animals, and the protocol was approved by the Institutional Animal Care and Use Committee in Daegu Haany University (Gyeongsan, Gyeongbuk, Korea) Approval No DHU2014-082. Eight mice in each group, with a total of six groups, were selected based on the body weights by ascending order after acclimatization: *Intact control* - Isocalorical maltose solution and distilled water administered mice, *EtOH control*: EtOH and distilled water administered mice, Silymarin group: EtOH and silymarin 250 mg/kg as reference drug treated mice, *HSCF 500*: mice administered EtOH and HSCF extracts at 500 mg/kg, HSCF 250: mice administered EtOH and HSCF extracts at 250 mg/kg, HSCF 125: mice administered EtOH and HSCF extracts at 125 mg/kg.

### Induction of EtOH-mediated subacute hepatic damage

Subacute EtOH-induced hepatotoxicity was induced by oral administration of EtOH (0.8 g/ml concentration; Merck, Darmstadt, Germany) at 5g per kg once a day for 14 days, at 1 hr before oral administration of each test substance, according to previous established methods^4,5,15^ with some modifications. In intact control mice, isocalorical maltose solution was orally administered instead of EtOH.

### Measurement of body weight and liver

Changes in body weight were measured at 1 day before initial test substance administration, the day of first test substance administration, and at 1, 7 10 and 14 days after initial HSCF or silymarin extracts administration using an automatic electronic balance (Precisa Instrument, Dietikon, Switzland). To reduce the individual differences, the body weight gains during 15 days of experiment were calculated as body weight on the last day of test substance administration – body weight on the first day of test substance administration. At sacrifice, the weights of the livers were measured (absolute wet-weights). To reduce the differences from individual body weights, relative weights were also calculated divided by body weight at sacrifice.

### Measurement of serum biochemistry

At sacrifice, about 1ml of venous blood was collected from the caudal vena cava under anesthesia with isoflurane (Hana Pharm. Co., Hwasung, Korea). All blood samples were centrifuged at 13,000 rpm, 4°C for 10 min using clotting activated serum tubes. Serum AST, ALT, albumin and ALP levels were detected using a blood biochemistrical autoanalyzer (Hemagen Analyst, Hemagen Diagnostic, Columbia, MA, USA). In addition, serum TG and *γ*-GTP levels were measured using another type of automated blood biochemistrical analyzer (SP-4410, Spotochem, Kyoto, Japan).

### Measurement of hepatic TG contents and TNF-*α* levels

To assess TG content, liver tissue (right lobes) was homogenized in an equal volume of normal saline and extracted with a mixture of chloroform and methanol (2:1) as described previously^36^. Zeolite (Sigma-Aldrich, St. Louise, MO, USA) was added to remove phospholipids. The resulting extract was dried under nitrogen and dissolved in Plasmanate (1ml; Sigma-Aldrich, St. Louise, MO, USA). TG were measured enzymatically using commercial kits (Kyowa Medex, Tokyo, Japan) as in previous studies^37^. Liver samples were disintegrated in 5 volumes of ice-cold radioimmunoprecipitation assay (RIPA) buffer. After incubation on ice for 30 min, samples were centrifuged twice at 20,000 × g for 15 min at 4°C. The supernatants were used for the assay. The contents of total protein were measured with the Lowry method^38^ using bovine serum albumin (Invitrogen, Carlsbad, CA, USA). The TNF-*α* levels were detected by enzyme-linked immunosorbent assay (ELISA) using a murine kit (BioSource International Inc., Camarillo, CA, USA) with a microplate reader (Tecan, Männedorf, Switzerland).

### Splenic cytokine content measurements

Splenic concentrations of TNF-*α*, IL-1β, and IL-10 were measured with a mouse TNF-*α* ELISA kit (BD Biosciences/ Pharmingen, San Jose, CA, USA), mouse IL-1β ELISA kit (Genzyme, Westborough, MA, USA) and mouse IL-10 ELISA kit (Genzyme, Westborough, MA, USA), respectively^39,40^. Approximately 10-15 mg of tissue samples were homogenized in a tissue grinder containing 1 ml of lysis buffer (PBS containing 2 mM PMSF and 1mg/ml of aprotinin, leupeptin, and pepstatin A)^41^. Analysis was performed with 100ml of standard (diluted in lysis buffer) or 10, 50, or 100 ml of tissue homogenate. Each sample was run in duplicate, and a portion of the sample was analyzed for protein.

### Determination of CYP450 2E1 Activity

Hydroxylation of p-nitrophenol to 4-nitrocatechol, a reaction catalyzed specifically by CYP2E1, was determined colorimetrically^4^. Liver tissue was homogenized in 0.15 KCl and was spun at 10,000×g for 30 min. Microsomes were isolated by further centrifugation at 105,000×g for 60 mins. For the assay, 300 ml of microsomal protein was incubated for 5 mins at 37°C, and absorbance at 535 nm was measured with 4-nitrocatechol (Sigma-Aldrich, St. Louise, MO, USA) as a standard using a UV/Vis spectrometer (OPTIZEN POP, Mecasys, Daejeon, Korea).

### Measurement of liver lipid peroxidation

Liver tissues were weighed and homogenized in ice-cold 0.01M Tris-HCl (pH 7.4), and then centrifuged at 12,000×g for 15 mins as described by Kavutcu et al^42^. The concentrations of liver lipid peroxidation were determined by estimating MDA using the thiobarbituric acid test at the absorbance of 525 nm and represented by nM of MDA/mg protein^43^. Total protein was measured by the Lowry method^38^.

### Measurement of hepatic antioxidant defense systems

Prepared homogenates were mixed with 0.1 ml of 25% trichloroacetic acid (Merck, West Point, CA, USA), and then centrifuged at 4,200 rpm for 40 min at 4 ºC. Glutathione (GSH) contents were measured at the absorbance of 412 nm using 2-nitrobenzoic acid (Sigma-Aldrich, St. Louise, MO, USA)^44^. Decomposition of H_2_O_2_ in the presence of catalase was performed at 240 nm^45^. Catalase activity was defined as the amount of enzyme required to decompose 1 nM of H_2_O_2_ per minute, at 25°C and pH 7.8. Measurements of SOD activities were made according to Sun et al.^46^.

### Reverse transcription-quantitative polymerase chain reaction (RT-qPCR)

RNA was extracted using Trizol reagent (Invitrogen, Carlsbad, CA, USA), according to the method described in previous studies5,15. The RNA concentrations and quality were determined with a CFX96^TM^ Real-Time System (Bio-Rad, Hercules, CA, USA). To remove contaminating DNA, samples were treated with recombinant DNase I (DNA-free; Ambion, Austin, TX, USA). RNA was reverse transcribed using the reagent High-Capacity cDNA Reverse Transcription Kit (Applied Biosystems, Foster City, CA, USA) according to the manufacturer’s instructions. Briefly, the cDNA strand was first synthesized from the total RNA and then the mixture of the primers and the cDNA products was amplified by PCR. The conditions of PCR amplification were 58°C for 30 mins, 94°C for 2 mins, 35 cycles of 94°C for 15 sec, 60°C for 30 sec, 68°C for 1 min, and then 72°C for 5 mins. Finally, the PCR products were separated on 0.8% agarose gel. Analysis was carried out using a gel imaging system (Bio-Rad, Hercules, CA, USA). Expression levels of SREBP-1c, SCD1, ACC1, FAS, PPAR*γ*, DGAT2, PPAR*α*, ACO, CPT1 and Nrf2 were calculated as a percentage relative to the intact group using β-actin RNA as the internal control. The sequences of the PCR oligonucleotide primers are listed in Table 1.

**Table 1.** Oligonucleotides primers used for RT-qPCR in this study

Target	5’ – 3’	Sequence	Gene ID
SREBP-1c	Forward	GATGTGCGAACTGGACACAG	6720
	Reverse	CATAGGGGGCGTCAAACAG	
SCD1	Forward	CCCCTGCGGATCTTCCTTAT	20249
	Reverse	AGGGTCGGCGTGTGTTTCT	
ACC1	Forward	CCATTGGTATTGGGGCTTAC	107476
	Reverse	CCCGACCAAGGACTTTGTTG	
FAS	Forward	GCTGCGGAAACTTCAGGAAAT	14102
	Reverse	AGAGACGTGTCACTCCTGGACTT	
PPARγ	Forward	AGTGGAGACCGCCCAGG	19016
	Reverse	GCAGCAGGTTGTCTTGGATGT	
DGAT2	Forward	AGTGGCAATGCTATCATCATCGT	67800
	Reverse	AAGGAATAAGTGGGAACCAGATCA	
PPARα	Forward	ATGCCAGTACTGCCGTTTTC	19013
	Reverse	GGCCTTGACCTTGTTCATGT	
ACO	Forward	GCCCAACTGTGACTTCCATT	74121
	Reverse	GGCATGTAACCCGTAGCACT	
CPT1	Forward	GCACTGCAGCTCGCACATTACAA	12894
	Reverse	CTCAGACAGTACCTCCTTCAGGAAA	
Nrf2	Forward	CGAGATATACGCAGGAGAGGTAAGA	18024
	Reverse	GCTCGACAATGTTCTCCAGCTT	
β-actin	Forward	CTGTCGAGTCGCGTCCA	11461
	Reverse	CTCGCGGGTGGACGCGACTCGACAG	

SREBP-1c, Sterol regulatory element-binding protein-1c; SCD1, Stearoyl-CoA desaturase 1; ACC1, Acetyl-CoA carboxylase 1; FAS, Fatty acid synthase; PPAR, Peroxisome proliferator-activated receptor; DGAT2, Diacylglycerol acyltransferase 2; ACO, Acyl-CoA oxidase; CPT1, Carnitine palmitoyltransferase 1; Nrf2, NF-E2-related factor-2

### Histopathological analysis

Left lateral lobes of the liver were fixed in 10% neutral buffered formalin (NBF), and embedded in paraffin, sectioned (3~4μm) and stained with Hematoxylin and eosin (H&E). Afterward, the histopathological profiles of each sample were observed under light microscope (Model 80i, Nikkon, Tokyo, Japan). For more detailed study, the number of hepatocytes, which occupied over 20% of lipid droplets in the cytoplasm, was calculated using an automated image analyzer (iSolution FL ver 9.1, IMT i-solution Inc., Vancouver, Canada). The value was reported as cells/1000 hepatocytes. The percentage of changed fatty regions (%/ mm^2^ of hepatic parenchyma) and the mean diameters of hepatocytes (μm/hepatocytes), with at least 10 hepatocytes per view field in the liver, were also calculated using an automated image analyzer in both the lateral and median lobes, according to the previously established method 47. The histopathologist was blinded to the group distribution when this analysis was conducted.

### Immunohistochemistry

After deparaffinization of the prepared hepatic histological paraffin sections, citrate buffer antigen (epitope) retrieval pretreatment was conducted as previously described^48,49^. Briefly, a water bath with staining dish containing 10 mM citrate buffer (pH 6.0) was preheated until the temperature reached 95-100°C. The slides were immersed in the staining dish and a lid was placed loosely on the staining dish. Incubation was performed for 20 min and the water bath was turned off. The staining dish was placed at room temperature and the slides were allowed to cool for 20 minutes. After epitope retrieval, sections were immunostained using avidin-biotin complex (ABC) methods (Table 2) for NT and 4-Hydroxynonenal (4-HNE) according to the previous study^48,50^. Briefly, endogenous peroxidase activity was blocked by incubation in methanol and 0.3% H_2_O_2_ for 30 minutes, and non-specific binding of immunoglobulin was blocked with normal horse serum blocking solution (Vector Lab., Burlingame, CA, USA. Dilution 1:100) for 1 hr in a humidity chamber. Primary antiserum (Table 2) was applied overnight at 4ºC in the humidity chamber, followed by incubation with biotinylated universal secondary antibody (Vector Lab., Dilution 1:50) and ABC reagents (Vectastain Elite ABC Kit, Vector Lab., Burlingame, CA, USA; Dilution 1:50) for 1 hr at room temperature in the humidity chamber. Finally, reaction with a peroxidase substrate kit (Vector Lab., Burlingame, CA, USA) was conducted for 3 min at room temperature. All sections were rinsed in 0.01M PBS 3 times between steps. The cells that showed stronger immunoreactivities in the cytoplasm with over 20% of the density against each antiserum as compared with intact control hepatocytes were regarded as positive immunoreactive. The numbers of NT- and 4-HNE-positive cells were measured for a total of 1000 hepatocytes using a digital image analyzer, according to previous reports^51-53^. The histopathologist was blinded to the group distribution when this analysis was performed.

**Table 2.** Primary antisera and detection kits used in Immunohistochemistry

Antisera or detection kits	Code	Source	Dilution
Primary antisera*			
Anti-4-Hydroxynonenal polyclonal antibody	ab46545	Abcam, Cambridge, UK	1:100
Anti-Nitrotyrosine polyclonal antibody	06-284	Millipore, Temecula, CA, USA	1:200
Detection kits			
Vectastain Elite ABC Kit	PK-6200	Vector Lab., Burlingame, CA, USA	1:50
Peroxidae substrate kit	SK-4100	Vector Lab., Burlingame, CA, USA	1:100

*All antiserum were diluted using 0.01M phosphate buffered saline (pH 7.2)

### Data Analysis

All numerical data were expressed as mean ± standard deviation (SD) of eight mice. Multiple comparison tests for different dose groups were conducted. Variance homogeneity was examined using the Levene test^54^. If the Levene test indicated no significant deviations from variance homogeneity, the obtained data were analyzed by one-way ANOVA test followed by least-significant differences multi-comparison (LSD) test to determine which pairs of group comparisons were significantly different. In the event of significant deviations from variance homogeneity in the Levene test, a non-parametric comparison test, Kruskal-Wallis H test, was conducted. When a significant difference was observed in the Kruskal-Wallis H test, the Mann-Whitney U (MW) test was conducted to determine the specific pairs of group comparison, which are significantly different. Statistical analyses were conducted using SPSS for Windows (Release 14.0K, IBM SPSS Inc., Armonk, NY, USA)^55^. In addition, the perpercent-point changes between intact vehicle and EtOH control were calculated to observe the severities of hepatic damages induced by 2 weeks of continuous oral treatment of EtOH in this study. The percent-point changes as compared with EtOH control and test substances treated mice were also calculated for understanding of the hepatoprotective effects of the test materials as in Equations 1 and 2, respectively, also according to our previous established method^56^.

Equation 1. Percent-point Changes as Compared with Intact Vehicle Control (%)

= ((Data of EtOH control – Data of intact control)/Data of intact control) × 100

Equation 2. Percent-point Changes as Compared with EtOH Control (%)

= ((Data for test substance administered group – Data of EtOH control)/Data for EtOH control) × 100.

## RESULTS

### Changes of the body weights

Significant (p<0.01 or p<0.05) decreases of body weight were detected from 7 days after EtOH administration in the EtOH control. The body weight gains during 15 days of experimentation were also significantly (p<0.01) decreased in the EtOH control as compared with the intact control. However, significant (p<0.01 or p<0.05) increases of body weights were observed from the 10th day of test substance administration in the mice treated with HSCF extracts at 500mg/kg and silymarin at 250 mg/kg, and from the 13th day in those treated with HSCF extracts at 250 and 125 mg/ kg as compared with the EtOH control. In addition, the body weight gains during 15 days of experiment were significantly (p<0.01) increased in HSCF and silymarin administered mice as compared with the EtOH control (Table 3).

**Table 3.** Body weight in mice with subacute EtOH-induced intoxication

Groups	Body weights (g)			Weight gains	Liver weight	
	Day - 1	Day 0 [A]*	Day 14 [B]*	[B-A]	absolute	relative
Controls						
Intact	21.96±1.58	19.86±1.73	23.21±2.04	3.35±0.87	0.881±0.095	3.801±0.351
EtOH	21.99±1.47	19.83±1.50	17.98±0.82^a^	- 1.85±0.94^a^	0.478±0.038^a^	2.661±0.207^a^
Silymarin	21.90±1.81	19.85±1.74	20.65±1.32^ab^	0.80±0.51^ab^	0.669±0.038^ab^	3.246±0.196^ab^
HSCF treated						
500 mg/kg	21.96±1.69	19.88±1.86	20.66±1.98^ab^	0.79±0.51^ab^	0.665±0.047^ab^	3.243±0.364^ab^
250 mg/kg	22.05±1.54	20.01±1.68	20.31±1.54^ab^	0.30±0.38^ab^	0.623±0.036^ab^	3.076±0.218^ab^
125 mg/kg	21.99±1.47	20.03±1.40	19.93±1.48^ac^	- 0.10±0.86^ac^	0.594±0.027^ab^	2.995±0.240^ac^

Values are expressed as Means ± SD of eight mice

*All animals were overnight fasted. Day 0 means the day of first test substance administration. Day 14 means 24 hrs after the last (14th) test substance administration

^a^ p<0.01 as compared with intact control by LSD test. ^b^ p<0.01 and ^c^ p<0.05 as compared with EtOH control by LSD test. EtOH, Ethanol; HSCF, Hoveniae Semen Cum Fructus extracts

### Changes in the liver weights

Significant (p<0.01) decreases of liver absolute wet- and relative weights were detected in EtOH control mice as compared with the intact control. However, these EtOH-induced decreases of liver absolute and relative weights were dose-dependently and significantly (p<0.01) inhibited by treatment with HSCF extracts at 500, 250 and 125 mg/kg as compared with the EtOH control mice. HSCF extracts at 500 mg/kg showed similar inhibitory effects on the EtOH-induced liver weight decreases as compared with silymarin at 250 mg/kg in this experiment (Table 3).

### Changes in the serum biochemistry

Significant (p<0.01) increases of serum AST, ALT, albumin, ALP, TG and *γ*-GTP levels were observed in the EtOH control as compared with the intact control. However, the serum chemistries were significantly (p<0.01) decreased by treatment with HSCF extracts at all dosages, and the effect was dose-dependent (Table 4).

**Table 4.** Changes in the serum biochemistry in Subacute EtOH-treated mice

Groups	Serum biochemistrical levels					
	AST (IU/l)	ALT (IU/l)	Albumin (g/dl)	ALP (IU/l)	TG (mg/dl)	γ-GTP (IU/l)
Controls						
Intact	90.00±21.78	46.38±13.96	3.88±0.82	66.75±14.42	148.88±28.03	2.00±0.76
EtOH	343.75±46.91^a^	158.13±37.16^c^	11.74±1.98^a^	246.38±27.84^a^	397.63±32.44^a^	7.50±1.60^a^
Silymarin	215.13±59.76^ab^	93.75±14.87^cd^	6.65±1.08^ab^	170.13±28.49^ab^	223.50±46.58^ab^	4.25±1.39^ab^
HSCF treated						
500 mg/kg	221.63±36.79^ab^	93.25±16.06^cd^	6.69±1.49^ab^	169.75±19.04^ab^	229.00±49.34^ab^	4.38±1.19^ab^
250 mg/kg	252.50±34.97^ab^	103.75±11.61^cd^	7.98±1.36^ab^	188.13±18.61^ab^	286.38±22.53^ab^	5.13±1.46^ab^
125 mg/kg	277.63±35.43^ab^	117.88±12.37^ce^	8.56±1.23^ab^	204.13±13.51^ab^	329.00±41.19^ab^	5.75±0.89^ab^

Values are expressed as Means ± SD of eight mice.

^a^ p<0.01 as compared with intact control by LSD test. ^c^ p<0.01 as compared with intact control by MW test. ^b^ p<0.01 as compared with EtOH control by LSD test; ^d^ p<0.01 and ^e^ p<0.05 as compared with EtOH control by MW test. EtOH, Ethanol; HSCF, Hoveniae Semen Cum Fructus extracts; ALP, Alkaline phosphatase; ALT, Alanine aminotransferase; AST, Aspartate aminotransferase; TG, Triglyceride; γ-GTP, γ-Glutamyl Transferase.

### Changes in the hepatic TG, TNF- *α* contents and CYP 450 2E1 activity

Significant (p<0.01) increases of liver TG contents were observed in the EtOH control as compared with the intact control mice. However, the liver TG contents were significantly (p<0.01) and dose-dependently decreased in HSCF extracts treated mice at all dosages. HSCF extracts at 500 mg/kg showed similar inhibitory effects on the EtOH-induced hepatic TG content elevation as compared with silymarin at 250 mg/kg (Table 5).

Significant (p<0.01) increases of liver TNF-*α* contents were observed in EtOH control as compared with intact control mice. However, the liver TNF-*α* contents were dose-dependently and significantly (p<0.01) decreased by treatment with HSCF extracts at all dosages. HSCF extracts at 500 mg/kg showed similar inhibitory effects on the EtOH-induced hepatic TNF-*α* elevation as compared with silymarin at 250 mg/kg in this study (Table 5).

Significant (p<0.01) increases of liver CYP450 2E1 activity and hydroxylation of p-nitrophenol to 4-nitrocatechol were observed in the EtOH control as compared with the intact control mice. However, the liver CYP450 2E1 activity was significantly (p<0.01) decreased by treatment with all dosages of HSCF extracts as compared with the EtOH control, dose-dependently. HSCF extracts at 500 mg/kg showed similar inhibitory effects on the EtOH-induced hepatic CYP450 2E1 activity increases as compared with silymarin at 250 mg/kg in this experiment (Table 5).

**Table 5.** Hepatic TG and TNF-α contents with hepatic CYP450 2E1 activity in subacute EtOH-treated mice

Groups	Hepatic contents		Hepatic CYP450 2E1 activity (4-nitrocatechol μM/min/mg protein)
	TG (mg/g tissue)	TNF-α (pg/mg protein)	
Controls			
Intact	18.94±3.51	27.70±15.99	1.91±0.49
EtOH	136.74±21.06^a^	90.46±17.04^a^	8.03±1.55^a^
Silymarin	79.93±16.24^ab^	52.81±13.12^ab^	4.40±1.49^ab^
HSCF treated			
500 mg/kg	81.19±17.88^ab^	53.63±12.32^ab^	4.45±1.10^ab^
250 mg/kg	98.73±15.49^ab^	61.89±17.33^ab^	5.34±0.88^ab^
125 mg/kg	110.26±17.64^ab^	67.60±13.40^ab^	6.06±0.97^ab^

Values are expressed means ±SD of ten mice.

^a^ p<0.01 as compared with intact control mice by LSD test. ^b^ p<0.01 as compared with EtOH control mice by LSD test. EtOH, Ethanol; HSCF, Hoveniae Semen Cum Fructus extracts; CY, Cytochrome; TG, Triglyceride;TNF, Tumor necrosis factor.

### Changes in the hepatic lipid peroxidation and antioxidant defense systems

Significant (p<0.01) increases in hepatic lipid peroxidation and increases of MDA contents in liver parenchyma were observed in the EtOH control mice as compared with the intact control mice. However, these increases in liver lipid peroxidation were significantly (p<0.01) and dose-dependently inhibited by treatment with HSCF extracts at 500, 250 and 125 mg/kg as compared with EtOH control mice. HSCF extracts at 500 mg/kg showed similar inhibitory effects on the EtOH-induced hepatic lipid peroxidation as compared with silymarin at 250 mg/kg in our experiment (Table 6).

Significant (p<0.01) decreases of hepatic GSH contents, SOD and CAT activities were detected in the EtOH control mice as compared with the intact control. However, hepatic antioxidant defense systems were significantly (p<0.01 or p<0.05) and dose-dependently enhanced by treatment with all dosages of HSCF extracts as compared with the EtOH control, resulting in significantly (p<0.01 or p<0.05) increased hepatic GSH contents, SOD and CAT activities as compared with EtOH control. Similar enhancement effects on the hepatic endogenous antioxidant defense systems were observed in mice treated with HSCF extracts at 500 mg/kg as compared with silymarin at 250 mg/kg (Table 6).

**Table 6.** Hepatic lipid peroxidation and antioxidant defense systems in subacute EtOH-treated mice

Groups	Malondialdehyde (nM/mg protein)	Glutathione (nM/mg protein)	Superoxide dismutase (U/mg protein)	Catalase (U/mg protein)
Controls				
Intact	1.47±0.66	41.08±12.23	416.67±104.78	251.99±49.21
EtOH	5.98±1.55^a^	6.27±2.14d	95.23±18.89^a^	85.39±16.77^d^
Silymarin	3.04±0.84^ab^	11.24±1.67^de^	197.15±61.73^ab^	177.36±32.38^de^
HSCF treated				
500 mg/kg	3.01±0.94^ab^	11.29±1.84^de^	199.87±85.74^ab^	178.87±18.32^de^
250 mg/kg	3.61±0.76^ab^	10.13±1.36^de^	186.96±79.78^ac^	170.08±22.94^de^
125 mg/kg	4.13±0.66^ab^	8.92±1.23^df^	173.68±18.98^ac^	158.35±22.90^de^

Values are expressed as Means ± SD of eight mice.

^a^ p<0.01 as compared with intact control by LSD test. ^d^ p<0.01 as compared with intact control by MW test. ^b^ p<0.01 and ^c^ p<0.05 as compared with EtOH control by LSD test. ^e^ p<0.01 and ^f^ p<0.05 as compared with EtOH control by MW test. EtOH, Ethanol; HSCF, Hoveniae Semen Cum Fructus extracts.

### Changes in the mRNA expression of hepatic lipogenic genes

To elucidate the molecular mechanism involved in the aggravation of EtOH-induced steatosis in HSCF extracts treated mice, the expression of genes regulating hepatic lipid synthesis was determined by quantitative RT-PCR, including SREBP-1c, SCD1, ACC1, FAS, PPAR*γ* and DGAT2 in the present study.

Hepatic SREBP-1c mRNA expression: In the EtOH control mice, significant (p<0.01) increases of hepatic SREBP-1c mRNA expression (SREBP-1c/β-actin mRNA) were observed as compared with the intact control mice. However, significant (p<0.01) dose dependent decreases of the hepatic SREBP-1c mRNA expression were demonstrated in mice treated with HSCF extracts at 500, 250 and 125 mg/kg as compared with the EtOH control mice. HSCF extracts at 500 mg/kg showed similar inhibitory effects on the EtOH-induced increases of hepatic SREBP-1c mRNA expression as compared with silymarin at 250 mg/kg in the present study (Table 7).

Hepatic SCD1 mRNA expression: In the EtOH control mice, significant (p<0.01) increases of hepatic SCD1 mRNA expression (SCD1/β-actin mRNA) were observed as compared with the intact control mice. However, significant (p<0.01) and dose-dependent decreases of the hepatic SREBP-1c mRNA expression were observed in mice treated with all three doses of HSCF extracts as compared with the EtOH control mice. Similar inhibitory effects on the hepatic SREBP-1c mRNA expression were demonstrated in mice treated with HSCF extracts at 500 mg/kg as compared with silymarin at 250 mg/kg in this study (Table 7).

Hepatic ACC1 mRNA expression: Significant (p<0.01) increases of liver ACC1 mRNA expression (ACC1/β-actin mRNA) were observed in the EtOH control as compared with the intact control mice. However, the hepatic ACC1 mRNA expression was significantly (p<0.01) and dose-dependently decreased by treatment with HSCF extracts at 500, 250 and 125 mg/kg, respectively. HSCF extracts at 500 mg/kg showed similar inhibitory effects on the EtOH-induced hepatic ACC1 mRNA expression increases as compared with silymarin at 250 mg/kg in this experiment (Table 7).

Hepatic FAS mRNA expression: In the EtOH control mice, significant (p<0.01) increases of hepatic FAS mRNA expression (FAS/β-actin mRNA) were observed as compared with the intact control mice. However, significant (p<0.01) and dose-dependent decreases of the hepatic FAS mRNA expression were observed with all three doses of HSCF extracts at 500, 250 and 125 mg/kg as compared with the EtOH control mice. HSCF extracts at 500 mg/ kg showed similar inhibitory effects on the EtOH-induced increases of hepatic FAS mRNA expression as compared with silymarin at 250 mg/kg (Table 7).

Hepatic PPAR*γ* mRNA expression: Significant (p<0.01) increases of liver PPAR*γ* mRNA expression (PPAR*γ*/ β-actin mRNA) were observed in the EtOH control as compared with the intact control mice. However, the hepatic PPAR*γ* mRNA expression was significantly (p<0.01 or p<0.05) decreased by treatment with all three doses of HSCF extracts. Similar inhibitory effects on the hepatic PPAR*γ* mRNA expression were observed in mice treated with HSCF extracts at 500 mg/kg as compared with silymarin at 250 mg/kg, in our study (Table 7).

Hepatic DGAT2 mRNA expression: In the EtOH control mice, significant (p<0.01) increases of hepatic DGAT2 mRNA expression (DGAT2/β-actin mRNA) were observed as compared with the intact control mice. However, significant (p<0.01) dose dependent decreases of the hepatic DGAT2 mRNA expression were observed in mice treated with HSCF extracts at 500, 250 and 125 mg/kg as compared with EtOH control mice. HSCF extracts at 500 mg/ kg showed similar inhibitory effects on the EtOH-induced hepatic DGAT2 mRNA expression increases as compared with silymarin at 250 mg/kg in our experiment (Table 7).

### Changes in the hepatic mRNA expression of genes involved in fatty acid oxidation

To elucidate the molecular mechanism involved in the aggravation of EtOH-induced steatosis in HSCF extracts treated mice, the expression of genes involved in fatty acid oxidation was also determined by quantitative RT-PCR, including PPAR*α*, ACO and CPT1 in the present study.

Hepatic PPAR*α* mRNA expression: Significant (p<0.01) decreases of hepatic PPAR*α* mRNA expression (PPAR*α*/ β-actin mRNA) were observed in the EtOH control as compared with the intact control mice. However, the hepatic PPAR*α* mRNA expression was significantly (p<0.01) and dose-dependently increased by treatment with all three doses of HSCF extracts at 500, 250 and 125 mg/kg. HSCF extracts at 500 mg/kg showed similar inhibitory effects on the EtOH-induced decreases of hepatic PPAR*α* mRNA expression as compared with silymarin at 250 mg/kg (Table 7).

Hepatic ACO mRNA expression: In the EtOH control mice, significant (p<0.01) decreases of hepatic ACO mRNA expression (ACO/β-actin mRNA) were observed as compared with the intact control mice. However, significant (p<0.01) dose dependent increases of the hepatic ACO mRNA expression were observed in mice treated with all three doses of HSCF extracts as compared with the EtOH control mice. Similar up-regulatory effects on the hepatic ACO mRNA expression were observed in mice treated with HSCF extracts at 500 mg/kg as compared to those treated with silymarin at 250 mg/kg (Table 7).

Hepatic CPT1 mRNA expression: In the EtOH control mice, significant (p<0.01) decreases of hepatic CPT1 mRNA expression (CPT1/β-actin mRNA) were observed as compared with the intact control mice. However, significant (p<0.01) and dose-dependent increases of the hepatic CPT1mRNA expression were observed in mice treated with HSCF extracts at 500, 250 and 125 mg/kg as compared with the EtOH control mice. HSCF extracts at 500 mg/kg showed similar inhibitory effects on the EtOH-induced hepatic CPT1 mRNA expression decreases as compared with silymarin at 250 mg/kg in our experiment (Table 7).

**Table 7.** Hepatic TG and TNF-α contents with hepatic CYP450 2E1 activity in subacute EtOH-treated mice

Groups	Controls			HSCF treated		
Genes	Intact	EtOH	Silymarin	500 mg/kg	250 mg/kg	125 mg/kg
Hepatic lipogenic genes						
SREBP-1c	1.00±0.19	3.79±0.96^d^	1.90±0.36^de^	1.91±0.42^de^	2.25±0.44^de^	2.41±0.30^de^
SCD1	1.01±0.11	3.84±1.01^d^	2.05±0.47^de^	2.16±0.47^de^	2.48±0.42^de^	2.81±0.25^de^
ACC1	0.99±0.10	2.32±0.43^d^	1.50±0.29^de^	1.51±0.18^de^	1.61±0.26^de^	1.82±0.18^de^
FAS	1.00±0.09	3.84±0.79^d^	2.13±0.48^de^	2.16±0.50^de^	2.46±0.47^de^	2.83±0.47^de^
PPARγ	1.00±0.06	5.34±1.51^d^	2.36±0.50^de^	2.47±0.54^de^	3.00±0.84^de^	3.51±0.69^df^
DGAT2	1.09±0.20	3.68±0.89^d^	2.09±0.42^de^	2.00±0.19^de^	2.43±0.60^de^	2.75±0.32^de^
Genes involved in fatty acid oxidation						
PPARα	0.96±0.19	0.41±0.16^a^	0.77±0.16^bc^	0.76±0.10^ac^	0.69±0.16^ac^	0.63±0.09^ac^
ACO	1.11±0.16	0.35±0.14^a^	0.70±0.14^ac^	0.69±0.11^ac^	0.62±0.09^ac^	0.54±0.09^ac^
CPT1	0.99±0.20	0.27±0.10^a^	0.63±0.15^ac^	0.65±0.11^ac^	0.52±0.12^ac^	0.48±0.11^ac^
Master transcription factor of antioxidant genes						
Nrf2	1.03±0.11	0.29±0.13^a^	0.61±0.17^ac^	0.63±0.12^ac^	0.58±0.10^ac^	0.47±0.07^ac^

Values are expressed as Means ± SD of eight mice.

a p<0.01 and b p<0.05 as compared with intact control by LSD test. d p<0.01 as compared with intact control by MW test. c p<0.01 as compared with EtOH control by LSD test. e p<0.01 and f p<0.05 as compared with EtOH control by MW test. EtOH, Ethanol; HSCF, Hoveniae Semen Cum Fructus extracts; RT-PCR = reverse transcription polymerase chain reaction; SREBP-1c = Sterol regulatory element-binding protein-1c; SCD1 = Stearoyl-CoA desaturase 1; ACC1 = Acetyl-CoA carboxylase 1; FAS= Fatty acid synthase; PPAR = Peroxisome proliferator-activated receptor; DGAT2 = Diacylglycerol acyltransferase 2; ACO = Acyl-CoA oxidase; CPT1 = Carnitine palmitoyltransferase 1; Nrf2 = NF-E2-related factor-2.

### Changes in the hepatic mRNA expression of Nrf2

To elucidate the molecular mechanism involved in the aggravation of EtOH-induced oxidative stress in HSCF extracts treated mice, the expression of the master transcription factor of antioxidant gene, Nrf2, was also determined by quantitative RT-PCR in the present study. Significant (p<0.01) decreases of hepatic Nrf2 mRNA expression (Nrf2/ β-actin mRNA) were demonstrated in the EtOH control as compared with the intact control mice. However, the hepatic Nrf2 mRNA expression was significantly (p<0.01) and dose-dependently increased by treatment with all three doses of HSCF extracts at 500, 250 and 125 mg/kg. HSCF extracts at 500 mg/kg showed similar inhibitory effects on the EtOH-induced decreases of hepatic Nrf2 mRNA expression as compared with silymarin at 250 mg/kg (Table 7).

### Effects on the liver histopathology

**Figure 1.**
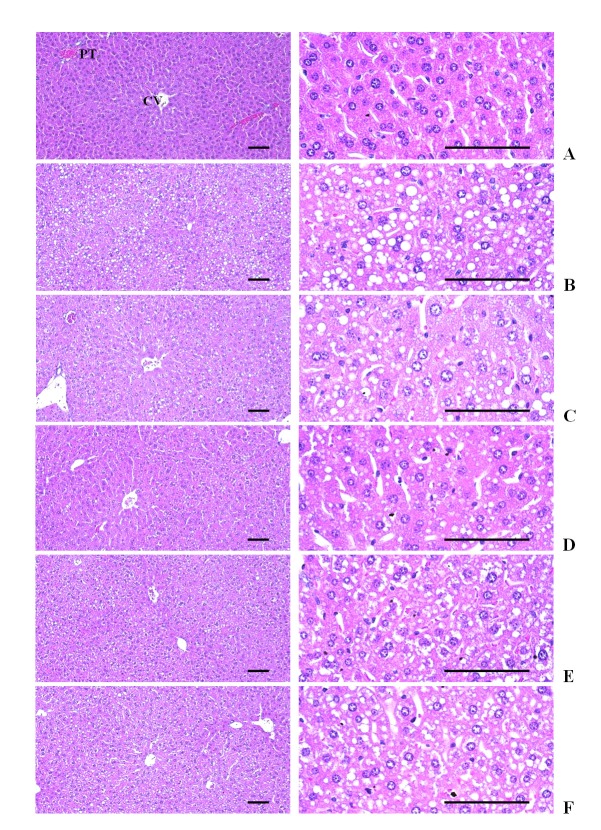
Representative histological images of the liver, taken from subacute EtOH-treated mice Severe deposition of lipid droplets in cytoplasm of hepatocytes, hepatosteatosis were observed in all EtOH-treated mice in the present study, and these EtOH-induced hepatosteatosis are re-confirmed with histomorphometry based on the number of changed fatty hepatocytes, mean diameters of hepatocytes and percentages of changed fatty regions, which were significantly increased in EtOH control mice as compared with intact control mice. However, this EtOH treatment-related histopathological hepatosteatosis was significantly inhibited by treatment with all three doses of HSCF extracts at 500, 250 and 125 mg/kg as compared with EtOH control mice, dose-dependently. Similar inhibitory effects on the EtOH-induced histopathological hepatosteatosis were demonstrated in mice treated with HSCF extracts at 500 mg/kg as compared with silymarin at 250 mg/kg in the present study. (A) Intact control, (B) EtOH control, (C) Silymarin control, (D) HSCF 500, (E) HSCF 250, (F) HSCF 125 EtOH, Ethanol; HSCF, Hoveniae Semen Cum Fructus extracts; CV, Central vein; PT, Portal triad Hematoxylin-eosin stain. Scale bars, 200μm.

Severe deposition of lipid droplets in the cytoplasm of hepatocytes and hepatosteatosis were observed in all EtOH-dosing groups in the present study. This EtOH-induced hepatosteatosis was re-confirmed with histomorphometry based on the number of changed fatty hepatocytes, mean diameters of hepatocytes and percentage of changed fatty regions, which were significantly (p<0.01) increased in the EtOH control mice as compared with the intact control mice. However, the EtOH treatment-related histopathological hepatosteatosis was significantly (p<0.01 or p<0.05) inhibited by treatment with all three doses of HSCF extracts at 500, 250 and 125 mg/kg as compared with the EtOH control mice, and the effect was dose-dependent. Similar inhibitory effects on the EtOH-induced histopathological hepatosteatosis were observed in mice treated with HSCF extracts at 500 mg/kg as compared to those treated with silymarin at 250 mg/kg in the present study (Table 8, Fig 1).

### Effects on the hepatic NT and 4-HNE-immunoreactivities

The immunoreactivities of NT as a marker of iNOS related oxidative stress^57^ and 4-HNE as a marker of lipid peroxidation^58^ in hepatic parenchyma were assessed to determine the liver oxidative stress.

**Figure 2.**
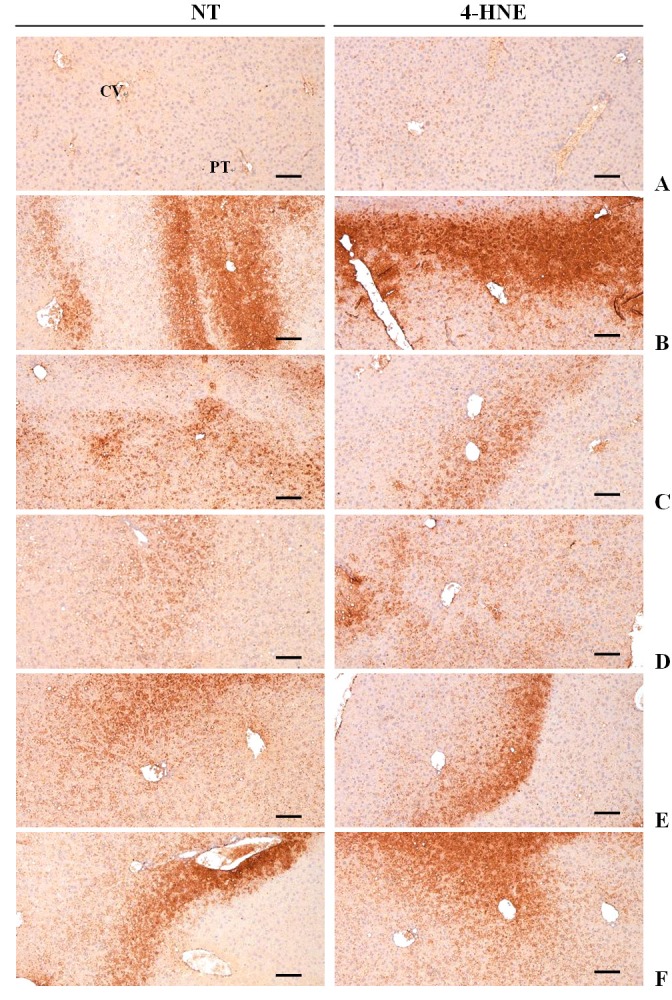
Representative images of NT and 4-HNE-immunoreactivities in the liver sections, taken from subacute EtOH-treated mice. Marked and significant increases of an iNOS related oxidative stress marker, nitrotyrosine-immunoreactive hepatocytes and also significant increases of a lipid peroxidation marker, 4-HNE-immunoreactive hepatocytes were observed in EtOH control mice as compared with intact control mice. HSCF extracts at 500, 250 and 125 mg/kg dose-dependently and significantly normalized these EtOH-related increases of NT- and 4-HNE-immunoreactive hepatocytes. HSCF extracts at 500 mg/kg showed similar inhibitory effects on the EtOH-induced hepatic NT- and 4-HNE-immunolabeled cell increases as compared with silymarin at 250 mg/kg in this study. (A) Intact control, (B) EtOH control, (C) Silymarin control, (D) HSCF 500, (E) HSCF 250, (F) HSCF 125 EtOH, Ethanol; HSCF, Hoveniae Semen Cum Fructus extracts; NT, Nitrotyrosine; 4-HNE, 4-hydroxynonenal; iNOS, inducible nitric oxide synthase, CV, Central vein; PT, Portal triad. ABC immunohistochemistrical methods. Scale bars, 200μm.

Changes in the NT-immunolabeled hepatocytes: Marked and significant (p<0.01) increases of an iNOS related oxidative stress marker, NT-immunoreactive hepatocytes, were observed in the EtOH control mice as compared with the intact control mice. HSCF extracts at 500, 250 and 125 mg/kg dose-dependently and significantly (p<0.01) normalized the EtOH-related increases of NT-immunoreactive hepatocytes. HSCF extracts at 500 mg/kg showed similar inhibitory effects on the EtOH-induced hepatic NT-immunolabeled cell increases as compared with silymarin at 250 mg/kg in this study (Table 8, Fig 2).

Changes in the 4-HNE-positive hepatocytes: Marked and significant (p<0.01) increases of a lipid peroxidation marker, 4-HNE-immunoreactive hepatocytes, were observed in the EtOH control mice as compared with the intact control mice. However, significant (p<0.01) decreases of the 4-HNE-immunopostive hepatocytes were demonstrated in mice treated with all three doses of HSCF extracts at 500, 250 and 125 mg/kg as compared with the EtOH control mice, and the effect was dose-dependent. HSCF extracts at 500 mg/kg showed similar inhibitory effects on the increases of the hepatic 4-HNE-immunolabeled cells induced by 2 weeks of continuous oral administration of EtOH as compared with silymarin at 250 mg/kg (Table 8, Fig 2).

**Table 8.** Hepatic TG and TNF-α contents with hepatic CYP450 2E1 activity in subacute EtOH-treated mice

Index	Fatty change regions (%)	Changed fatty hepatocyte numbers (cells/1000 hepatocyte)	Mean hepatocyte diameters (μm)	Numbers NT- immunolabeled cells (cells/1000 hepatocyte)	Numbers 4-HNE immunopositive cells (cells/1000 hepatocyte)
Groups					
Controls					
Intact	8.44±2.78	86.75±31.11	18.05±2.42	44.00±18.08	77.50±27.67
EtOH	77.93±10.79^a^	743.75±138.164^d^	32.52±3.25^a^	562.63±92.50^d^	685.13±112.77^a^
Silymarin	36.37±10.93^ac^	387 00±102.73^de^	23.91±2.83^ac^	228.50±68.96^de^	195.88±61.02^ac^
HSCF treated					
500 mg/kg	35.74±8.05^ac^	365.25±119.80^de^	23.28±3.26^ac^	215.50±82.25^de^	171.88±47.04^bc^
250 mg/kg	52.88±10.89^ac^	513.63±101.61^de^	26.10±3.64^ac^	319.88±63.24^de^	263.50±76.53^ac^
125 mg/kg	59.01±13.03^ac^	557.63±99.95^df^	27.88±2.92^ac^	430.00±66.80^de^	472.25±84.84^ac^

Values are expressed as Means ± SD of eight mice.

a p<0.01 and b p<0.05 as compared with intact control by LSD test. d p<0.01 as compared with intact control by MW test. c p<0.01 as compared with EtOH control by LSD test. e p<0.01 and f p<0.05 as compared with EtOH control by MW test. EtOH, Ethanol; HSCF, Hoveniae Semen Cum Fructus extracts; 4-HNE, 4-Hydroxynonenal; NT, Nitrotyrosine

## DISCUSSION

Alcoholic liver disease remains one of the most common causes of chronic liver disease in the world^59^. Alcoholic fatty liver is the earliest and most common response of the liver to heavy alcohol use, and it is a precursor of more severe forms of liver injury^5,15,16,60^. Accumulated evidence has demonstrated that oxidative stress, abnormal cytokine production, and steatosis play important etiological roles in the pathogenesis of alcoholic liver disease. Therefore, agents that have antioxidant, anti-inflammatory and anti-steatosis properties, particularly anti-TNF production and decreasing lipid accumulation, represent a promising therapeutic intervention for alcoholic liver disease^4,5,7^. Various antioxidant-based pharmacological effects of HSCF extracts have been reported including anti-adipogenic^31^ and hepatoprotective^34,35^ effects. However, it seems that more systemic evaluation of the hepatoprotective effects of HSCF extract with molecular targets is needed. In the present study, the beneficial potential of HSCF extract on the subacute EtOH-induced hepatic damages in mice was systemically evaluated as well as the corresponding potent anti-oxidant, anti-inflammatory and anti-steatosis mechanisms.

The body weight decrease after EtOH treatment was considered a result of the direct toxicity of EtOH and/or indirect toxicity related to liver damage. The body weight can also decrease due to malnutrition, secondary to food intake decreases^61,62^. Therefore, the increased body weight and gains detected in the silymarin group and HSCF extracts treated group are considered indirect evidence of hepatoprotective effects as compared with the EtOH control, since body weight is considered a putative indicator of health. In addition, dose-dependent inhibitory effects on the EtOH-induced liver weight decreases following treatment with HSCF extracts were also considered evidence that HSCF extracts have hepatoprotective effects against acute EtOH intoxication. In chronic alcoholics, the liver weight is generally decreased due to necrotic and inflammatory processes that occur in the hepatic parenchyma and the substitution of hepatic parenchyma with lipids 63-66. This is also the case in acute EtOH-induced liver damaged mice67. HSCF extracts at 500 mg/kg showed similar inhibitory effects on the EtOH-induced liver weight decreases as compared with silymarin at 250 mg/kg in this experiment.

Generally, AST, ALT, albumin, *γ*-GTP and ALP are used as serum markers to represent various types of liver damage 68. These markers were markedly elevated following EtOH-induced hepatic damages in previous reports^69,70^ and also in this experiment. In addition, serum TG levels are generally increased with EtOH-induced hepatic damage due to decreased TG utilization in hepatocytes^71,72^. Therefore, it is considered evidence that HSCF extracts have favorable hepatoprotective effects against EtOH-induced liver injuries because marked inhibition of the EtOH-induced serum AST, ALT, albumin, ALP, *γ*-GTP and TG levels, and hepatic TG contents were dose-dependently improved in these groups as compared with the EtOH control mice in the present study. HSCF extracts at 500 mg/kg showed similar inhibitory effects on the EtOH-induced serum AST, ALT, albumin, ALP, TG and *γ*-GTP elevation as compared with silymarin at 250 mg/kg.

Abnormal metabolism of cytokines, especially TNF-*α*, is another major feature of alcoholic liver disease^4,7^. It was initially observed that cultured monocytes from alcoholic hepatitis patients spontaneously produced TNF-*α* and produced significantly more TNF-*α* in response to a lipopolysaccaride stimulus than control monocytes^73^. Subsequently, earlier researchers demonstrated that anti-TNF antibody prevented liver injury in alcohol-fed rats and mice lacking the TNF-type I receptor also did not develop alcoholic liver injury^74,75^. Consistent with chronic alcohol effects, increased hepatic TNF-*α* production by acute EtOH exposure has recently been reported^4,7,76^. *In vitro* studies demonstrated that silymarin inhibited Kupffer cell functions and TNF-*α* production in lipopolysaccaride-stimulated RAW264.7 cells^77,78^. Our results also showed that 2 weeks of continuous subacute EtOH administration enhanced hepatic TNF-*α* production. In vivo HSCF extracts administration dose-dependently attenuated this increased TNF-*α* production, similar to silymarin at 250 mg/kg, suggesting that the hepatoprotective effects of HSCF extracts on EtOH-induced subacute hepatic damages may be mediated by anti-inflammatory effects through suppression of the hepatic TNF-*α* production.

Although there are many potential sources of ROS in response to EtOH exposure, CYP450 2E1 is one of the major sites involved in ROS production in the liver in response to alcohol4. It has been reported that long-term alcohol exposure increased CYP450 2E1 activities^76,79^. Furthermore, investigations using CYP450 2E1 inhibitors, including diallyl sulfide or chlormethiazole, have shown that inhibition of CYP450 2E1 activity inhibits alcohol-induced liver injury, indicating the importance of CYP450 2E1 in alcohol- induced ROS accumulation and liver injury^80,81^. Similarly, genetic overexpression of CYP450 2E1 in the liver causes enhanced alcohol-induced liver injury in mice^82^. To investigate the possible mechanisms by which HSCF extracts attenuated subacute EtOH-induced liver injury, we first evaluated the effect of HSCF extracts on CYP450 2E1 enzymatic activity in response to acute EtOH exposure. Our study indicated that 2 weeks of continuous oral administration of EtOH increased hepatic CYP450 2E1 activity, but this increase of CYP450 2E1 activity was dose-dependently diminished by treatment with HSCF extracts. HSCF extracts at 500 mg/kg showed similar inhibitory effects on the EtOH-induced hepatic CYP450 2E1 activity increases as compared with silymarin at 250 mg/kg.

Considerable experimental and clinical evidence has contributed to support a key role of oxidative stress in the pathophysiological processes of liver injury related to excessive alcohol consumption^83,84^. The metabolism of EtOH gives rise to the generation of excess amounts of ROS and has a detrimental effect on the cellular antioxidant defense system^85,86^ that leads to hepatic cellular necrosis, inflammation and steatohepatitis^64,87^. Thus, numerous interventions have been put forward to counteract the vulnerability of the liver to oxidative challenges during alcohol consumption by reinforcing the endogenous antioxidant defense system^86,88^. Lipid peroxidation is an autocatalytic mechanism leading to oxidative destruction of cellular membranes^89,90^. Such destruction can lead to cell death and to the production of toxic and reactive aldehyde metabolites called free radicals, with MDA as the most important^91,92^. It is known that ROS leads to oxidative damage of biological macromolecules, including lipids, proteins, and DNA9^1,93^, and oxidative stress influences body adipocytes, resulting in decreases in body fat mass and related body weight decreases^94^. MDA is a terminal product of lipid peroxidation. So the content of MDA can be used to estimate the extent of lipid peroxidation^91^. Marked increases of liver MDA contents have been observed in alcoholic rodents^4,5,7,15,64^, and liver MDA content was increased in this study by treatment with EtOH. GSH is a representative endogenous antioxidant that prevents tissue damage by keeping the ROS at low levels and at certain cellular concentrations, and is accepted as a protective antioxidant factor in tissues^95^. SOD is one of the antioxidant enzymes that contributes to enzymatic defense mechanisms, and catalase is an enzyme that catalyzes the conversion of H_2_O_2_ to H_2_O^96^. The decrease of antioxidant enzyme activities such as SOD and catalase, and GSH contents may be indicative of the failure to compensate for the oxidative stress induced by EtOH^64,85-87^. In this experiment, the hepatic antioxidant defense system was dose-dependently enhanced by treatment with HSCF extracts at 500, 250 and 125 mg/kg as compared with the EtOH control, along with up-regulation of Nrf2, a master transcription factor of antioxidant genes^17,19^, which was down-regulated by EtOH supply. HSCF extracts at 500 mg/kg showed similar inhibitory effects on the EtOH-induced hepatic lipid peroxidation, and enhancement effects on the hepatic endogenous antioxidant defense systems as compared with silymarin at 250 mg/kg. This suggests that the hepatoprotective effects of HSCF extracts against EtOH intoxication are mediated by augmentation of the hepatic antioxidant defense system, which may be mediated by Nrf2 activation and related inhibitory effects on lipid peroxidation.

There are multiple mechanisms underlying EtOH-induced development of fatty liver. Enhanced lipogenesis and impaired fatty-acid oxidation have long been proposed as important biochemical mechanisms underlying the development of alcoholic fatty liver^5,15^. Previous studies demonstrated that EtOH administration activates SREBP- 1c and its target genes like SCD1, ACC1 and FAS, which promote *de novo* fatty-acid synthesis^5,15,23^. SREBP-1cnull mice fed EtOH by intragastric infusion for 4 weeks showed significantly lower TG concentration than that in wild typed mice^97^. In this experiment, EtOH treatment also significantly up-regulated the hepatic SREBP-1c mRNA expression, and its target genes – FAS, SCD1 and ACC1. However, all dosages of HSCF extracts dose-dependently down regulated the hepatic mRNA expression of SREBP- 1c, SCD1, ACC1 and FAS. This suggests that the hepatoprotective effects of HSCF extracts against EtOH-induced hepatic steatosis are mediated by down regulation of SREBP-1c and its target genes, FAS, SCD1 and ACC1. HSCF extracts at 500 mg/kg showed similar inhibitory effects on the EtOH-induced increases of hepatic lipogenic genes - SREBP-1c, FAS, SCD1 and ACC1 mRNA expression as compared with silymarin at 250 mg/kg.

PPAR*γ* and DGAT2 are involved in TG synthesis^5,15,22-24^. DGAT is involved in TG synthesis in the liver, and the levels of DGAT1 and DGAT2 mRNAs were increased in response to EtOH^5,15^. PPAR*γ* is a member of the nuclear receptor superfamily of ligand-activated transcription factors that regulate the expression of genes associated with lipid metabolism. Adenovirus-mediated delivery of PPAR*γ* to hepatocytes leads to fatty liver, and PPAR*γ* RNA interference is reported to decrease hepatic TG levels^22,24^. PPAR*γ* and DGAT are significantly up-regulated after acute EtOH administration and are involved in EtOH-induced fatty liver in mouse^5,15,23^. In this study, hepatic mRNA levels of both PPAR*γ* and DGAT2 were up-regulated by EtOH stimulation. HSCF extracts at 500, 250 and 125 mg/kg significantly and dose-dependently impaired the elevation of these genes, similar to silymarin at 250 mg/kg, well corresponding to the results of hepatic and serum TG levels. These results suggested that oral treatment of HSCF extracts dose-dependently inhibits hepatic lipogenesis in response to EtOH by suppressing genes related to TG synthesis.

In addition to increased lipogenesis, decreased fatty acid metabolism also contributes to EtOH-induced fatty liver^5,15,23,60^. PPAR*α* and its target genes, including ACO and CPT1, are involved in fatty-acid β-oxidation^5,15,25^. Administration of Wy14643, a PPAR*α* agonist, prevented fatty liver in mice fed EtOH for 4 weeks^98,99^. In this experiment, subacute treatment of EtOH 5 g/kg decreased the expression of these genes and impaired fatty-acid β-oxidation in the liver. However, HSCF extracts at 500, 250 and 125 mg/ kg up-regulated the hepatic mRNA expression of PPAR*α* and its target genes, including ACO and CPT1, similar to silymarin at 250 mg/kg. Oral treatment of HSCF extracts at dose levels of 500, 250 and 125 mg/kg not only down regulated the expression of genes related to fatty-acid and TG synthesis, but also increased fatty acid metabolism through up-regulation of genes involved in fatty-acid β-oxidation in the liver.

Acute or chronic alcohol consumption can cause severe histopathological liver injury^100^. Alcohol is known to impair fat oxidation and to stimulate lipogenesis in the liver^101-103^. Thus, alcohol consumption can lead to the development of hepatic steatosis^104^. In this experiment, severe deposition of lipid droplets in the cytoplasm of hepatocytes and hepatosteatosis were also observed in all EtOH treated mice. This EtOH-induced hepatosteatosis was re-confirmed with histomorphometry based on the number of changed fatty hepatocytes, mean diameters of hepatocytes and percentages of changed fatty regions, which were significantly increased in EtOH control mice as compared with intact control mice in the left lateral lobes. However, this EtOH treatment-related histopathological hepatosteatosis was significantly and dose-dependently inhibited by treatment of HSCF extracts at 500, 250 and 125 mg/kg, similar to silymarin 250 mg/kg, as compared with EtOH control mice in this experiment. These findings are considered direct evidence that HSCF extracts have favorable hepatoprotective effects against EtOH-induced hepatic steatosis.

NT is a product of tyrosine nitration mediated by reactive nitrogen species such as peroxynitrite anion and nitrogen dioxide. It is detected in a large number of pathological conditions including EtOH-induced liver damages, and is considered a marker of nitric oxide-dependent, reactive nitrogen species-induced nitrative stress^51,57,105^. Most studies on alcoholic hepatic steatosis have focused on the ability of EtOH to shift the redox state in the liver and to inhibit fatty acid oxidation^101,106^. Indeed, previous studies have shown the repression of some enzymes involved in fatty acid oxidation and induction of lipogenic enzymes in EtOH-fed animals^102,103^. Sustained exposure to ROS leads to prolonged oxidative stress and increases of NT^107,108^. In this experiment, marked and significant increases of NT-immunoreactive cells were observed in the hepatic tissues of EtOH control mice as compared with intact control mice, but they were significantly reduced by treatment of HSCF extracts at 500, 250 and 125 mg/kg, dose-dependently, similar to silymarin at 250 mg/kg. It is suggested that HSCF extracts favorably inhibit iNOS related oxidative stress and protect against hepatocyte necrotic changes from EtOH at dose levels of 500, 250 and 125 mg/kg.

4-HNE is an *α*, β-unsaturated hydroxyalkenal which is produced by lipid peroxidation in cells. It is considered a possible causal agent of numerous diseases, such as chronic inflammation, neurodegenerative diseases, adult respiratory distress syndrome, atherogenesis, diabetes and different types of cancer^58,109,110^. Sustained exposure to EtOH mediated ROS leads to prolonged oxidative stress, which promotes lipid peroxidation and generation of reactive aldehydes, such as 4-HNE^107,111^. In the present study, marked and significant increases of 4-HNE-positive cells were also observed in the left lateral hepatic lobes of EtOH control mice as compared with intact control mice, but they were significantly and dose-dependently normalized by treatment of all dosages of HSCF extracts, similar to silymarin at 250 mg/kg. This corresponded to the results of NT-immunolabeled cells and is considered as direct evidence that HSCF extracts effectively inhibited lipid peroxidation and the formation of 4-HNE to protect against hepatocyte necrotic changes from EtOH.

Results corresponding to previous reports^4,5,7,15^ regarding marked decreases of body and liver weights, increases of serum AST, ALT, Albumin, *γ*-GTP and TG levels, hepatic TG contents, TNF-*α* level, CYP450 2E1 activity and mRNA expression of hepatic lipogenic genes (SREBP- 1c, SCD1, ACC1, FAS, PPAR*γ* and DGAT2), decreases mRNA expression of genes involved in fatty acid oxidation (PPAR*α*, ACO and CPT1) or master transcription factor of antioxidant gene (Nrf2) were observed with histopathological changes related to hepatosteatosis (noticeable increases of the percentages of changed fatty regions, the number of changed fatty hepatocytes and mean hepatocyte diameters) and increases of NT and 4-HNE-immunolabelled hepatocytes, following continuous oral administration of EtOH for 2 weeks in the present study. Also, the destruction of hepatic antioxidant defense systems (the increase of hepatic lipid peroxidation, increase of liver MDA contents, and decreases of GSH contents, SOD and CAT activities) were demonstrated in EtOH control mice as compared with intact control. However, these EtOH treatment related liver inflammatory damages, steatosis, increases of mRNA expression of hepatic lipogenic genes, decreases of mRNA expression of genes involved in fatty acid oxidation, and destruction of antioxidant defense systems, may be mediated by down-regulation of Nrf2, which was markedly and dose-dependently inhibited by pretreatment of HSCF extracts at 500, 250 and 125 mg/kg. The overall effects of HSCF extracts at 500 mg/kg were similar to those of silymarin at 250 mg/kg in this experiment.

## CONCLUSION

This study found that oral administration of 500, 250, and 120 mg/kg of HSCF favorably protected against liver damages from subacute mouse EtOH intoxication. Hoveniae Semen Cum Fructus extract demonstrated potent anti-inflammatory and anti-steatosis properties through augmentation of the hepatic antioxidant defense system, mediated by Nrf2 activation and down-regulation of the mRNA expression of hepatic lipogenic genes or up-regulation of the mRNA expression of genes involved in fatty acid oxidation. Hoveniae Semen Cum Fructus extract seems to be a new potent hepatoprotective agent or ingredient for liver diseases, with less toxicity.
